# Evidence of trospium’s ability to mitigate cholinergic adverse events related to xanomeline: phase 1 study results

**DOI:** 10.1007/s00213-023-06362-2

**Published:** 2023-04-10

**Authors:** Alan Breier, Stephen K. Brannan, Steven M. Paul, Andrew C. Miller

**Affiliations:** 1 Department of Psychiatry, Indiana University School of Medicine, Indianapolis, IN USA; 2 Karuna Therapeutics, Boston, MA USA

**Keywords:** KarXT, Xanomeline, Trospium, Muscarinic receptor agonist, Tolerability, Schizophrenia, Pharmacokinetics, Healthy volunteers, Phase 1

## Abstract

**Rationale:**

The M_1_/M_4_ preferring muscarinic receptor agonist xanomeline demonstrated antipsychotic and procognitive effects in patients with Alzheimer’s disease or schizophrenia in prior studies, but further clinical development was limited by cholinergic adverse events (AEs). KarXT combines xanomeline with the peripherally restricted muscarinic receptor antagonist trospium with the goal of improving tolerability and is in clinical development for schizophrenia and other neuropsychiatric disorders.

**Objective:**

Test the hypothesis that trospium can mitigate cholinergic AEs associated with xanomeline.

**Methods:**

Healthy volunteers enrolled in this phase 1 (NCT02831231), single-site, 9-day, double-blind comparison of xanomeline alone (*n* = 33) versus KarXT (*n* = 35). Rates of five prespecified cholinergic AEs (nausea, vomiting, diarrhea, excessive sweating, salivary hypersecretion) were compared between treatment arms. Vital signs, electrocardiograms (ECGs), safety laboratory values, and pharmacokinetic (PK) analyses were assessed. A self-administered visual analog scale (VAS) and clinician-administered scales were employed.

**Results:**

Compared with xanomeline alone, KarXT reduced composite incidences of the five a priori selected cholinergic AEs by 46% and each individual AE by ≥ 29%. There were no episodes of syncope in KarXT-treated subjects; two cases occurred in the xanomeline-alone arm. The rate of postural dizziness was 11.4% in the KarXT arm versus 27.2% with xanomeline alone. ECG, vital signs, and laboratory values were not meaningfully different between treatment arms. The VAS and clinician-administered scales tended to favor KarXT. PK analysis revealed that trospium did not affect xanomeline’s PK profile.

**Conclusions:**

Trospium was effective in mitigating xanomeline-related cholinergic AEs. KarXT had an improved safety profile compared with xanomeline alone.

**Supplementary information:**

The online version contains supplementary material available at 10.1007/s00213-023-06362-2.

## Introduction

Numerous lines of investigation have implicated muscarinic cholinergic systems in the pathophysiology of psychotic disorders and led to targeting muscarinic acetylcholine receptors for innovative therapeutics for these illnesses (Felder et al. 2000; Paul et al. 2022; Raedler et al. 2007). Xanomeline, a potent M_1_/M_4_-preferring muscarinic receptor agonist (Thorn et al. 2019), showed antipsychotic efficacy in previous placebo-controlled clinical trials in patients with Alzheimer’s disease (AD) (Bodick et al. 1997) and schizophrenia (Shekhar et al. 2008). Despite its promising efficacy profile, the development of xanomeline was discontinued because of significant levels of cholinergic adverse events (AEs), namely nausea, vomiting, diarrhea, excessive sweating, and salivary hypersecretion (Bodick et al. 1997; Shekhar et al. 2008). In addition to activating central M_1_ and M_4_ receptors, which are believed to mediate xanomeline’s efficacy for psychosis and cognitive impairment (Felder et al. 2018; Moran et al. 2019), xanomeline also stimulates muscarinic acetylcholine receptors localized in peripheral tissue, which likely mediate these cholinergic AEs. A strategy to mitigate xanomeline’s cholinergic AEs was necessary to further develop this agent for neuropsychiatric disorders.

KarXT combines xanomeline with trospium, a pan-muscarinic receptor antagonist that is restricted to the periphery (Staskin et al. 2010). Trospium is approved by the U.S. Food and Drug Administration and is a widely used and generally well-tolerated treatment for overactive bladder (Indevus Pharmaceuticals 2012). It was hypothesized that the peripheral muscarinic receptor blocking action of trospium would mitigate xanomeline-related cholinergic AEs without interfering with its central mechanism of action.

We present the results of a phase 1 healthy volunteer study (NCT02831231) that was designed to test the ability of trospium to mitigate xanomeline-related cholinergic AEs. The rates of five prespecified cholinergic AEs (nausea, vomiting, diarrhea, excessive sweating, and salivary hypersecretion) were compared between xanomeline plus trospium and xanomeline alone. In addition, vital signs, electrocardiograms (ECGs), and safety laboratory values were assessed, and a self-administered visual analog scale (VAS) and clinician-administered scales were employed. Also, a pharmacokinetic (PK) analysis of xanomeline was conducted to determine if the addition of trospium affected xanomeline blood levels. This trial paved the way for future studies of KarXT in patients with schizophrenia and other neuropsychiatric disorders.

## Methods

Healthy volunteers aged 18−60 years were enrolled in this 9-day, single-site, double-blind, placebo-controlled phase 1 trial. Subjects were required to be in good general health, able and willing to use an acceptable form of contraception if of childbearing potential, and able to give informed consent. Exclusion criteria included history or presence of clinically significant disease, history of alcohol or drug abuse, and history of suicidal ideation. Full inclusion and exclusion criteria are reported in Supplementary Information 1. The study was conducted in September–October 2016 at a single site (Medpace, Inc., Cincinnati, Ohio).

After a 21-day screening period, eligible subjects were randomized 1:1 to receive either KarXT or xanomeline alone. The dose of xanomeline was 75 mg given three times per day and the dose of trospium was 20 mg given twice per day. There was a 2-day lead-in period during which subjects received only placebo or trospium followed by 7 days of xanomeline in addition to either placebo or trospium. Safety was assessed using spontaneous reports of AEs including the five prespecified cholinergic AEs (nausea, vomiting, diarrhea, excessive sweating, and salivary hypersecretion). A treatment-emergent AE (TEAE) was defined as any AE that happened for the first time after dosing of study drug or existed before but worsened in severity or relationship to the study drug after dosing. AEs were summarized by system organ class and preferred term using the Medical Dictionary for Regulatory Activities (Version 19.0). The site investigator assessed AEs for severity and as being either related or unrelated to study drug.

Vital signs, ECGs, and safety laboratory tests were also obtained. In addition, self-rated VAS for severity of the cholinergic AEs scored from 0 mm (none) to 100 mm (extreme) were administered three times per day for the 7-day active xanomeline treatment phase (total of 21 administrations). The mean weekly maximum composite VAS scores for nausea, vomiting, diarrhea, sweating, and excess salivation (considering all events together) for the 7-day treatment period were a priori determined to be used in the analysis. This variable was calculated by dividing the sum of the maximum VAS scores recorded for each cholinergic AE on each of Day 3 through Day 9 by the total number of maximum VAS scores recorded. Subjects who completed the 7-day active phase and remained in the study through Day 9 had a maximum of 35 VAS scores that contributed to this composite score (seven daily maximum scores for each of five cholinergic AEs). If a subject prematurely discontinued from the active phase prior to Day 9, then the mean weekly maximum composite VAS scores were calculated using the scores that were available. A full schedule of assessments in the study is reported in Supplementary Information 2.

The following scales were administered by clinicians: Postoperative Nausea and Vomiting Impact Scale (Myles and Wengritzky 2012) assessed nausea and vomiting, Unified Parkinson’s Disease Rating Scale (Ramaker et al. 2002) assessed excess salivation, Hyperhidrosis Disease Severity Scale (Solish et al. 2007) measured excess sweating, and Bristol Stool Form Scale (O'Donnell et al. 1990) assessed a wide range of stool consistencies, including diarrhea. Total saliva volume (mL) was also collected. Lastly, the PK profile (maximum velocity, area under the curve [AUC]) of xanomeline was determined at Day 3 and Day 9.

A key endpoint for this study was mean weekly maximum composite VAS score, which was calculated and compared between treatment arms (KarXT vs xanomeline alone). Supportive analyses included the mean weekly maximum individual VAS scores for each of the five cholinergic adverse events. In addition, clinician-administered scale scores were compared between groups. The incidence and percentage difference in rate of cholinergic TEAEs for the KarXT group compared with xanomeline alone was included as a post hoc analysis. The “evaluable” population was used to assess the key and supportive endpoints and consisted of all subjects who received at least one dose of xanomeline and at least one post-randomization VAS rating starting from the day of randomization. Assuming that the mean difference between treatment groups for the key endpoint (mean weekly maximum composite VAS score) would be 15 mm on the VAS (standard deviation for the difference: 20 mm), a sample size of 60 subjects (30 subjects/treatment arm) was estimated to provide approximately 81% power to detect a significant difference between treatment arms using a two-sided significance level of 5%. Statistical testing was based on a two-sample t-test (continuous variables), a chi-squared test (categorical variables), or a Fisher's exact test (small sample sizes) and not adjusted for multiplicity for baseline demographic and characteristic data. All other data regarding safety and AEs were summarized descriptively for each treatment arm using the evaluable population.

PK parameters were summarized by treatment arm and study day (with standard noncompartmental methods) using the PK population, which included subjects who had at least one measurable post-dose PK concentration of xanomeline or trospium.

The study was conducted in accordance with the Declaration of Helsinki, applicable laws and regulations, and Good Clinical Practice Guidelines. The protocol and informed consent document were approved by an institutional review board (Schulman Institutional Review Board, Cincinnati, Ohio) prior to study initiation. All subjects provided written informed consent prior to the initiation of any study procedures.

## Results

**Fig. 1:**
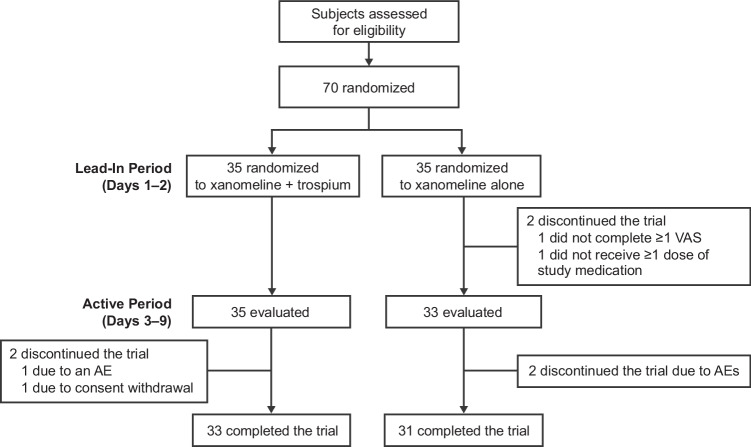
Subject disposition. Abbreviations: *AE* adverse event; *VAS* visual analog scale

A total of 70 healthy volunteers were randomized to xanomeline alone (*n* = 35) or KarXT (*n* = 35). The analyses were conducted in the evaluable population, which was defined as all subjects who received ≥ 1 dose of xanomeline and ≥ 1 VAS score for each cholinergic AE on ≥ 1 day of the active-treatment phase (Day 3 through Day 9): xanomeline alone (*n* = 33) and KarXT (*n* = 35). Two subjects who had been assigned to the xanomeline arm dropped out during the 2-day trospium/placebo lead-in phase; of these, one subject did not complete at least one VAS and the other subject did not receive at least one dose of xanomeline prior to discontinuing the study. As such, these two subjects were excluded from the evaluable sample, which comprised 68 subjects (Fig. 1). Baseline demographics and characteristics are summarized in Table 1.

**Table 1:** Baseline demographics and characteristics

Characteristic	Xanomeline Alone (*n* = 33)	Xanomeline + Trospium (*n* = 35)
Age, mean (SD), years	34.8 (8.8)	40.9 (12.3)^a^
Sex, n (%)		
Male	21 (63.6)	27 (77.1)
Female	12 (36.4)	8 (22.9)
Race, n (%)		
White	9 (27.3)	13 (37.1)
Black or African American	23 (69.7)	21 (60.0)
Other	1 (3.0)	1 (2.9)
Ethnicity, n (%)		
Hispanic or Latino	0	0
Not Hispanic or Latino	33 (100)	35 (100)
Weight, mean (SD), kg	88.4 (16.6)	87.8 (15.8)
BMI, mean (SD), kg/m^2^	29.1 (5.0)	28.8 (5.0)

*BMI* body mass index; *df *degrees of freedom; *SD* standard deviation

^a^*p* = 0.024 versus xanomeline alone (t = 2.32; df = 66)

An overview of all AEs is shown in Table 2. In all categories, KarXT had a lower incidence of reported AEs than xanomeline alone (Table 2). The proportion of subjects reporting any TEAE was 81.8% on xanomeline alone and 65.7% on KarXT. Table 3 contains the incidence and percent of cholinergic TEAEs for xanomeline alone and KarXT. There was a 46% reduction in the incidence of any cholinergic AEs reported by subjects treated with KarXT compared with xanomeline alone (34.3% vs. 63.6%, respectively). KarXT was associated with a 59% reduction in sweating. In addition, there was a reduction of ≥ 29% in the incidence of each of the four other individual cholinergic AEs by KarXT compared with xanomeline alone. The total number of cholinergic AEs was approximately half for KarXT (*n* = 34) compared with xanomeline alone (*n* = 64). There was an incidence of 33.3% (placebo) and 5.7% (trospium only) for the xanomeline-alone and KarXT treatment groups, respectively, for any cholinergic AE during the 2-day lead-in period, which is suggestive of background rates.

**Table 2:** Overview of all AEs

Characteristic, No. subjects (% subjects) [No. events]	Xanomeline Alone (*n* = 33)	Xanomeline + Trospium (*n* = 35)
TEAE		
≥ 1	27 (81.8) [108]	23 (65.7) [73]
None	6 (18.2)	12 (34.3)
Maximum severity of TEAEs		
Mild	22 (66.7)	20 (57.1)
Moderate	5 (15.2)	3 (8.6)
Severe	0	0
Any clinically significant TEAE^a^	5 (15.2) [5]	3 (8.6) [6]
Any study drug–related TEAE	23 (69.7) [92]	18 (51.4) [57]
Maximum severity of study drug–related TEAEs		
Mild	19 (57.6)	15 (42.9)
Moderate	4 (12.1)	3 (8.6)
Severe	0	0
Any SAE	0	0
Any AE leading to discontinuation	2 (6.1) [2]	1 (2.9) [1]
Any study drug–related AE leading to discontinuation	1 (3.0) [1]	0

*AE* adverse event; *No.* number; *SAE* serious adverse event; *TEAE* treatment-emergent adverse event

^a^Defined as moderate or higher severity

**Table 3:** Incidence of cholinergic TEAEs

Preferred term, No. subjects (% subjects)	Xanomeline Alone (*N* = 33)	Xanomeline + Trospium (*N* = 35)	Difference in Incidence Rate
Any cholinergic TEAE^a^	21 (63.6)	12 (34.3)^b^	46%
Sweating	16 (48.5)	7 (20.0)^c^	59%
Salivary hypersecretion	12 (36.4)	9 (25.7)	29%
Nausea	8 (24.2)	6 (17.1)	29%
Diarrhea	7 (21.2)	2 (5.7)	73%
Vomiting	5 (15.2)	2 (5.7)	62%

*df *degrees of freedom; *No.* number; *TEAE* treatment-emergent adverse event

^a^TEAE was defined as any adverse event (Medical Dictionary for Regulatory Activities [Version 19.0]) that happened for the first time or worsened in severity after the dosing of study drug. Nausea, vomiting, diarrhea, excessive sweating, and salivary hypersecretion were prespecified cholinergic adverse events

^b^*p* = 0.016 versus xanomeline alone (X^2^ = 5.86, df = 1 [any cholinergic TEAE])

^c^*p* = 0.013 versus xanomeline alone (X^2^ = 6.16, df = 1 [sweating])

Results of additional key AEs (n [%] No. of events) were syncope: 2 (6.1%) 2 versus 0 (0.0) 0 and postural dizziness: 9 (27.3%) 15 versus 4 (11.4%) 5 (xanomeline alone vs. KarXT, respectively). Three subjects experienced AEs leading to discontinuation: one subject randomized to KarXT experienced a mild AE of elevated blood pressure during the lead-in phase that was considered unrelated to study drug and discontinued the study on Day 3 prior to the first xanomeline dose; one subject randomized to xanomeline alone experienced presyncope of moderate intensity unrelated to study drug; and one subject randomized to xanomeline alone experienced syncope of moderate intensity that the investigator considered related to study drug. There were no clinically meaningful differences between the two groups for any vital signs, ECG, or safety laboratory values, including on renal or liver function tests.

For the VAS, the weekly mean ± SD total cholinergic composite scores (mm) were 3.82 ± 5.50 for xanomeline alone (median 1.49, range 0.0–23.6) and 2.29 ± 6.65 for KarXT (median 0.00, range 0.0–32.8), with a mean difference (95% confidence interval) of -1.54 (-4.50, 1.43). Individual cholinergic AE VAS scores were directionally consistent with the TEAE data (presented above) favoring KarXT. Results of clinician-administered scales were as follows: nausea and vomiting (per Postoperative Nausea and Vomiting Scale assessment) were seen less frequently in the KarXT group than the xanomeline-alone group (14.7% vs. 25.0% and 2.9% vs. 15.6%, respectively; Supplementary Information 3, Table S1). Unified Parkinson’s Disease Rating Scale assessed excess salivation, which was seen less frequently in the KarXT group compared with the xanomeline-alone groups (3.2% vs. 16.1%, respectively, at Day 9; Supplementary Information 3, Table S2). Hyperhidrosis Disease Severity Scale measured excess sweating, which was less common in the KarXT group compared with the xanomeline alone group (3.2% vs. 19.4%, respectively, at Day 9; Supplementary Information 3, Table S3). The Bristol Stool Form Scale showed no discernible difference between the groups (Supplementary Information 3, Table S4). Total saliva volume (mL) was less for KarXT compared with xanomeline alone (51.4 vs. 75.1, respectively).

**Fig. 2:**
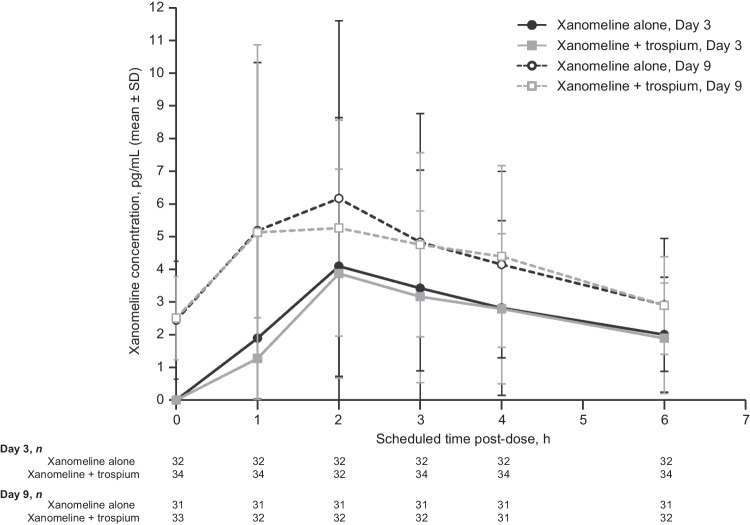
Pharmacokinetic concentration–time profile for xanomeline by treatment arm and study day. Abbreviation: *SD* standard deviation

Results of the population PK analysis for xanomeline levels are shown in Table 4 and Fig. 2. Xanomeline PK parameters were generally similar for the two treatment arms on Days 3 and 9, indicating that trospium did not measurably affect xanomeline blood levels or its PK profile.

**Table 4:** Pharmacokinetic parameters

Parameter	Xanomeline Alone		Xanomeline + Trospium	
	*n*	Mean (SD)	*n*	Mean (SD)
Day 3				
C_max_, ng/mL	32	4.42 (4.74)	34	3.98 (3.12)
T_max_, h	32	2.65 (0.93)	34	2.62 (1.00)
AUC_0-last_, h*ng/mL	32	15.31 (16.22)	34	13.95 (10.82)
T_1/2_, h	11^a^	3.53 (1.18)	17^a^	3.28 (1.21)
Day 9				
C_max_, ng/mL	31	6.78 (6.03)	32	6.23 (5.67)
T_max_, h	31	2.12 (1.09)	32	2.13 (0.91)
AUC_0-last_, h*ng/mL	31	26.08 (20.38)	32	25.33 (16.70)
T_1/2_, h	21^a^	4.69 (2.12)	21^a^	4.44 (2.45)

*AUC*_*0-last*_ area under the concentration–time curve from time 0 to time of last measurable concentration; *C*_*max*_ maximum observed concentration; *SD* standard deviation; *T*_*1/2*_ apparent terminal half-life; *T*_*max*_ time to reach maximum observed concentration

^a^Only reported for subjects who exhibited a terminal elimination phase in their concentration versus time profiles. The short duration of the dosing interval may not have allowed for adequate characterization of the terminal elimination phase in some subjects

## Discussion

The results from this phase 1 multi-dose study demonstrate that trospium is effective at mitigating xanomeline-related cholinergic AEs. Our study showed that KarXT reduced overall cholinergic AEs by 46% compared with xanomeline alone. In addition, KarXT was associated with a 59% reduction in excessive sweating and a reduction of ≥ 29% in the incidence of each of the five a priori selected individual cholinergic AEs of interest.

ECGs, vital signs, and laboratory values were similar between the treatment arms. There were no episodes of syncope in KarXT-treated subjects (two cases occurred in the xanomeline-alone arm) and postural dizziness was noted at lower rates in the KarXT arm (11.4%) compared with xanomeline alone (27.2%). The PK analysis revealed that trospium did not affect the PK profile of xanomeline. Overall, adding trospium to xanomeline (KarXT) decreased cholinergic AEs compared with xanomeline alone.

The self-rated cholinergic VAS scores favored KarXT compared with xanomeline alone. However, this analysis was limited because the scores were restricted to the low end of the scale with cholinergic weekly composites score (mean ± SD) of 3.82 ± 5.50 mm (xanomeline alone) and 2.29 ± 6.65 mm (KarXT) on a 100-mm scale. The low scoring indicated that the VAS was not a sensitive metric of AEs with xanomeline, likely due to the episodic nature and relatively low total event rate for individual cholinergic AEs and the relatively high number of VAS administrations (21) over the study week, which were then averaged as weekly scores. In addition, the clinician-administered AE instruments tended to favor KarXT. Total salivary volume was higher in the xanomeline-alone group versus the KarXT group, which is consistent with incidence rates of salivary hypersecretion TEAEs (lower in KarXT subjects). This difference in salivary volume also presents a peripheral pharmacodynamic marker that trospium is blocking the activation of muscarinic receptors by xanomeline.

Limitations of the current study include the lack of sensitivity of the VAS measures used, objective measures of peripheral cholinergic activity, and assessment of cholinergic AEs prior to dosing rather than 2–3 h post dose (corresponding to the time needed to reach maximum concentration (Table 4)). While the study made a placebo-based comparison of KarXT (xanomeline + trospium) to xanomeline + placebo, the study did not have a placebo-only arm. Also, a longer treatment period may inform time-dependent decreases in cholinergic AEs that were observed in the schizophrenia trial (Brannan et al. 2021), which was completed following the trial described herein. Lastly, although rates of diarrhea as a reported cholinergic AE were reduced, group differences in diarrhea were not observed with the Bristol Stool Form Scale. This inconsistency may have resulted from a combination of low diarrhea rates overall, all events being mild, and the fact that the Bristol Stool Form Scale encompasses a wide range of stool consistencies; thus, infrequent instances of mild diarrhea could fall into any of several categories within the scale and group differences would be undetectable with the current sample size.

The results presented here are from a trial in healthy volunteers designed to show differences between xanomeline and KarXT and test the hypothesis that trospium would reduce cholinergic AEs. There are three previously published placebo-controlled studies in patients with AD and schizophrenia that provide pertinent comparative information related to the five prespecified cholinergic AEs contained in this paper (Table 5): a phase 2 trial in 343 patients with AD (*n* = 87 assigned to the high-dose arm of 225 mg/day of xanomeline alone) (Bodick et al. 1997); a small phase 2 trial in 20 patients with schizophrenia (*n* = 10 assigned to 225 mg/day of xanomeline alone) (Shekhar et al. 2008); and a phase 2 trial in 182 patients with schizophrenia of KarXT (*n* = 90 assigned to KarXT, xanomeline dose up to 250 mg/day; trospium 60 mg/day) (Brannan et al. 2021), which was conducted subsequent to the current phase 1 trial. Xanomeline doses were similar in all four trials. As shown in Tables 3 and 5, KarXT was associated with substantially lower rates of cholinergic AEs compared with historical trials of xanomeline alone in AD and schizophrenia. In the phase 2 trial of KarXT in schizophrenia, cholinergic AE rates ranged from -2% to 13% on a placebo-adjusted basis (calculated as the incidence in the xanomeline-alone and KarXT treatment arms minus the incidence rate in the placebo treatment arms), compared with 7% to 72% placebo-adjusted in the AD trial (Table 5). This comparison of trial results must be treated with caution given the significant differences between these trials – trial duration, subject numbers, populations, and ages (e.g., the AD trial included patients with advanced ages [> 59 years] and included a 6-month trial duration). However, the results across these three phase 2 trials demonstrate substantially higher rates of cholinergic AEs in the xanomeline-alone trials compared with the KarXT trial.

**Table 5:** Comparison of placebo-adjusted rates^a^ (%) of five cholinergic AEs from three clinical trials

Preferred term (%)	Xanomeline Alone AD (Bodick et al. 1997) (*n* = 87^b^)	Xanomeline Alone Schizophrenia (Shekhar et al. 2008) (*n* = 10)	Xanomeline + Trospium Schizophrenia Phase 2 (Brannan et al. 2021) (*n* = 89^c^)
Sweating	71	20	1
Salivary hypersecretion	24	10	0
Nausea	32	30	13
Diarrhea	7^d^	20	-2
Vomiting	33	50	5

*AD* Alzheimer’s disease; *AE* adverse event

^a^Calculated as the incidence in the xanomeline-alone and KarXT treatment arms minus the incidence rate in the placebo treatment arms

^b^High-dose treatment arm (225 mg/day)

^c^89 of the 90 subjects assigned to xanomeline + trospium qualified for the safety population

^d^AE was reported as “fecal incontinence”

The rates of cholinergic AEs (Table 5) were numerically lower in the phase 2 schizophrenia trial (Brannan et al. 2021) compared with the KarXT arm in the current study (Table 3). The phase 2 study incorporated an initial dose titration phase that may have contributed to these lower rates and included a parallel placebo arm that enhanced interpretation of drug-related events. In addition, patients with chronic schizophrenia and extensive lifetime exposures to psychotropic medication may display better psychotropic drug tolerability than healthy volunteers, which may have also contributed to the lower rates of cholinergic AEs in the phase 2 trial.

AEs are a critically important factor in medication adherence, tolerability, and safety. Trospium was shown to be effective in mitigating xanomeline-related cholinergic AEs, permitting further development of this compound for neuropsychiatric disorders. KarXT is currently under investigation in phase 3 studies for patients with schizophrenia and in trials of patients with dementia-related psychosis. Future studies are needed to further elucidate the efficacy and safety of KarXT as a potential antipsychotic and procognitive treatment for patients with schizophrenia and AD.

## Supplementary information

Below is the link to the electronic supplementary material.

Supplementary file1 (PDF 156 KB)

Supplementary file2 (PDF 201 KB)

Supplementary file3 (PDF 241 KB)

## Data Availability

Data will be made available from the corresponding author on reasonable request, subject to review.

## References

[CR1] Allergan, Inc (2012) Sanctura (trospium chloride). Prescribing information. https://www.accessdata.fda.gov/drugsatfda_docs/label/2012/021595s009lbl.pdf

[CR2] Bodick NC, Offen WW, Levey AI, Cutler NR, Gauthier SG, Satlin A, Shannon H, Tollefson G, Rasmussen K, Bymaster FP, Hurley DJ, Potter WZ, Paul SM (1997). Effects of xanomeline, a selective muscarinic receptor agonist, on cognitive function and behavioral symptoms in Alzheimer disease. Arch Neurol.

[CR3] Brannan SK, Sawchak S, Miller AC, Lieberman JA, Paul SM, Breier A (2021). Muscarinic cholinergic receptor agonist and peripheral antagonist for schizophrenia. N Engl J Med.

[CR4] Felder CC, Bymaster FP, Ward J, DeLapp N (2000). Therapeutic opportunities for muscarinic receptors in the central nervous system. J Med Chem.

[CR5] Felder CC, Goldsmith PJ, Jackson K, Sanger HE, Evans DA, Mogg AJ, Broad LM (2018). Current status of muscarinic M1 and M4 receptors as drug targets for neurodegenerative diseases. Neuropharmacology.

[CR6] Moran SP, Maksymetz J, Conn PJ (2019). Targeting muscarinic acetylcholine receptors for the treatment of psychiatric and neurological disorders. Trends Pharmacol Sci.

[CR7] Myles PS, Wengritzky R (2012). Simplified postoperative nausea and vomiting impact scale for audit and post-discharge review. Br J Anaesth.

[CR8] O'Donnell LJ, Virjee J, Heaton KW (1990). Detection of pseudodiarrhoea by simple clinical assessment of intestinal transit rate. BMJ.

[CR9] Paul SM, Yohn SE, Popiolek M, Miller AC, Felder CC (2022). Muscarinic acetylcholine receptor agonists as novel treatments for schizophrenia. Am J Psychiatry.

[CR10] Raedler TJ, Bymaster FP, Tandon R, Copolov D, Dean B (2007). Towards a muscarinic hypothesis of schizophrenia. Mol Psychiatry.

[CR11] Ramaker C, Marinus J, Stiggelbout AM, Van Hilten BJ (2002). Systematic evaluation of rating scales for impairment and disability in Parkinson's disease. Mov Disord.

[CR12] Shekhar A, Potter WZ, Lightfoot J, Lienemann J, Dubé S, Mallinckrodt C, Bymaster FP, McKinzie DL, Felder CC (2008). Selective muscarinic receptor agonist xanomeline as a novel treatment approach for schizophrenia. Am J Psychiatry.

[CR13] Solish N, Bertucci V, Dansereau A, Hong HC, Lynde C, Lupin M, Smith KC, Storwick G, Canadian Hyperhidrosis Advisory C (2007). A comprehensive approach to the recognition, diagnosis, and severity-based treatment of focal hyperhidrosis: recommendations of the Canadian Hyperhidrosis Advisory Committee. Dermatol Surg.

[CR14] Staskin D, Kay G, Tannenbaum C, Goldman HB, Bhashi K, Ling J, Oefelein MG (2010). Trospium chloride has no effect on memory testing and is assay undetectable in the central nervous system of older patients with overactive bladder. Int J Clin Pract.

[CR15] Thorn CA, Moon J, Bourbonais CA, Harms J, Edgerton JR, Stark E, Steyn SJ, Butler CR, Lazzaro JT, O'Connor RE, Popiolek M (2019). Striatal, hippocampal, and cortical networks are differentially responsive to the M4- and M1-muscarinic acetylcholine receptor mediated effects of xanomeline. ACS Chem Neurosci.

