# Supplementary material for: Evidence of trospium’s ability to mitigate cholinergic adverse events related to xanomeline: phase 1 study results
Source: Psychopharmacology (Berl). 2023 Apr 10;240(5):1191–8. doi: 10.1007/s00213-023-06362-2 (PMC10102054; doi:10.1007/s00213-023-06362-2)
Supplement: Supplementary file 1 — Supplementary file1 (PDF 156 KB) [file 213_2023_6362_MOESM1_ESM.pdf]

Article Title: Evidence of trospium’s ability to mitigate cholinergic adverse events related to xanomeline:

phase 1 study results

Journal Name: *Psychopharmacology*

Author Names: Alan Breier, Stephen K. Brannan, Steven M. Paul, Andrew C. Miller

Corresponding Author: Andrew C. Miller; Karuna Therapeutics, Boston, MA, USA;
amiller@karunatx.com, (857) 449-2244

SUPPLEMENTARY INFORMATION 1: FULL INCLUSION AND EXCLUSION CRITERIA

SUPPLEMENTARY INFORMATION 1: FULL INCLUSION AND EXCLUSION CRITERIA

Inclusion Criteria

To be enrolled in the study, subjects were required to meet the following criteria:

1. Age 18 to 60 at screening;
2. Female subjects must be post-menopausal (≥2 years prior to dosing) or surgically sterile or have a negative pregnancy test and agree to use an acceptable form of contraception (hormonal or double barrier method of birth control; abstinence) from screening until 30 days after last dose. If oral contraceptives are used, the subject must be on a stable dose for ≥6 months;
3. Male subjects must agree to use a barrier method of birth control (condom with spermicide; vasectomy with spermicide), not impregnate a sexual partner during the study or for 90 days after the final dose of study drug, and refrain from sperm donation for 90 days after the final dose of study drug;
4. Be in good general health;
5. Able to give informed consent and understand verbal instructions;
6. Agree to avoid any over-the-counter medications during the course of the study (acetaminophen as needed ≤500 mg daily allowed); and
7. Willing to comply with study requirements and restrictions

Exclusion Criteria

Subjects who met any of the following criteria were excluded from the study:

1. History or presence of clinically significant cardiovascular, pulmonary, hepatic, renal, hematologic, gastrointestinal, endocrine, immunologic, dermatologic, neurologic, oncologic, or psychiatric disease or any other condition that, in the opinion of the Investigator, would jeopardize the safety of the subject or the validity of the study results;

2. Body mass index <18 or >40 kg/m^2;
3. History of, or high risk of, urinary retention, gastric retention, or narrow-angle glaucoma;
4. History of alcohol or drug abuse within the last 24 months, or current abuse as determined by urine toxicology screen or breathalyzer test;
5. Any history of suicidal ideation;
6. Clinically significant abnormal finding on the physical examination, medical history, electrocardiogram, or clinical laboratory results at screening;
7. Participation in another clinical trial within 90 days prior to the first dose of study drug;
8. Use of any prescription medication within 14 days prior to dosing besides the investigational product and hormonal contraceptives;
9. Use of any vitamins, herbs, supplements, or over-the-counter medications within 1 week of enrollment, and during the course of the trial. Specifically, subjects may not take diphenhydramine for 1 week prior and during the course of the study;
10. Pregnant or breastfeeding;
11. Use of any tobacco products within the past 30 days; or
12. Previous positive test for HIV 1 and/or 2, or hepatitis B or C, or a positive test obtained at screening
