# Supplementary material for: Evidence of trospium’s ability to mitigate cholinergic adverse events related to xanomeline: phase 1 study results
Source: Psychopharmacology (Berl). 2023 Apr 10;240(5):1191–8. doi: 10.1007/s00213-023-06362-2 (PMC10102054; doi:10.1007/s00213-023-06362-2)
Supplement: Supplementary file 2 — Supplementary file2 (PDF 201 KB) [file 213_2023_6362_MOESM2_ESM.pdf]

Article Title: Evidence of trospium's ability to mitigate cholinergic adverse events related to xanomeline: phase 1 study results

Journal Name: *Psychopharmacology*

Author Names: Alan Breier, Stephen K. Brannan, Steven M. Paul, Andrew C. Miller

Corresponding Author: Andrew C. Miller; Karuna Therapeutics, Boston, MA, USA;
amiller@karunatx.com, (857) 449-2244

SUPPLEMENTARY INFORMATION 2: SCHEDULE OF ASSESSMENTS

	Screening	Clinic admission	Lead-in phase		Xanomeline + trospium or xanomeline + placebo								Follow-up
Day	-21 to -1	0	1	2	3	4	5	6	7	8	9	10	24 ± 1
Procedure													
Informed consent signed	X												
Eligibility review and confirmation	X	X											
Medical history	X												
Physical examination	X	X										X	X
Serum pregnancy test (females of childbearing potential only)	X											X	
Urine pregnancy test (females of childbearing potential only)		X											
Urine drug screen and breathalyzer test	X	X											
HIV and viral hepatitis screen	X												
Vital signs and orthostatic blood pressure[a]	X	X	X	X	X	X	X	X	X	X	X	X	X
Clinical laboratory and urinalysis	X	X			X			X				X	X
Height and body weight[b]	X	X										X	
12-lead ECG (prior to & 2 hours post morning dose)[c]	X		X		X							X	X
Medication history	X	X											
Randomization			X										
Administer study drug[d]			X	X	X	X	X	X	X	X	X		
Pharmacokinetic sampling[e]		X	X	X	X	X	X		X		X	X	
Cholinergic AE assessments (patient VAS)[f]			X	X	X	X	X	X	X	X	X	X	
Completion of clinician-administered scales (PONV, UPDRS [salivation subscale], HDSS, Bristol Stool Form Scale)[g]			X	X	X	X	X	X	X	X	X	X	
Salivation volume measurement[h]			X	X	X	X	X	X	X	X	X	X	
Spontaneously reported AE assessments	X	X	X	X	X	X	X	X	X	X	X	X	X

Abbreviations: *AE* adverse event; *BMI* body mass index; *ECG* electrocardiogram; *HDSS* Hyperhidrosis Disease Severity Scale; *PK* pharmacokinetic; *PONV* Postoperative Nausea and Vomiting; *UPDRS* Unified Parkinson's Disease Rating Scale; *VAS* visual analog scale

[a]Vital signs, including systolic blood pressure, diastolic blood pressure, heart rate, and body temperature, and orthostatic blood pressure were tested once at screening and once on Days 0 through 9, Day 10 prior to discharge, and Day 24. On Days 1 through 9, they were taken prior to morning dose and 2 hours (±15 min) post morning dose. Only vital signs were taken on Day 24. When vital signs and orthostatic blood

pressure were scheduled at the same time as blood draws, vital signs and blood pressure were obtained before and as close to the scheduled blood draw as possible and were obtained after a 12-lead ECG

[b]Height and weight were taken at screening to derive BMI. Weight only was taken on Day 0 to derive BMI and confirm continued eligibility and on Day 10

[c]Twelve-lead ECG was taken once at screening and Days 1, 3, 10, and 24. On Days 1 and 3, 12-lead ECG was taken prior to and 2 hours (±15 min) post morning dose

[d]Study drug was administered 3 times per day at 6 ± 1-hour intervals

[e]Serial PK samples were collected on Days 1, 3, and 9 following the morning dose. On Days 2, 4, 5, 7, and 10, a single sample was collected prior to the morning dose to monitor trough study drug concentrations

[f]Cholinergic AEs were assessed using VAS instruments 3 times per day prior to each dose of study drug

[g]Clinician-administered scales were completed once per day between the afternoon and evening doses

[h]Salivation volume was assessed prior to morning dose on Days 1 and 3, once per day on Days 1 through 9 between the afternoon and evening doses, and once on Day 10 prior to discharge
