# Supplementary material for: Evidence of trospium’s ability to mitigate cholinergic adverse events related to xanomeline: phase 1 study results
Source: Psychopharmacology (Berl). 2023 Apr 10;240(5):1191–8. doi: 10.1007/s00213-023-06362-2 (PMC10102054; doi:10.1007/s00213-023-06362-2)
Supplement: Supplementary file 3 — Supplementary file3 (PDF 241 KB) [file 213_2023_6362_MOESM3_ESM.pdf]

Article Title: Evidence of trospium's ability to mitigate cholinergic adverse events related to xanomeline: phase 1 study results

Journal Name: *Psychopharmacology*

Author Names: Alan Breier, Stephen K. Brannan, Steven M. Paul, Andrew C. Miller

Corresponding Author: Andrew C. Miller; Karuna Therapeutics, Boston, MA, USA; amiller@karunatx.com, (857) 449-2244

SUPPLEMENTARY INFORMATION 3: EXPLORATORY ENDPOINTS

Table S1 Patient-reported nausea and vomiting during the active phase (Days 3–9) as assessed by the Postoperative Nausea and Vomiting scale

Table S2 Patient-reported salivation during the active phase (Days 3–9) as assessed by the Unified Parkinson's Disease Rating Scale salivation subscale

Table S3 Patient-reported sweating during the active phase (Days 3–9) as assessed by the Hyperhidrosis Disease Severity Scale

Table S4 Bristol Stool Form Scale

Table S1 Patient-reported nausea and vomiting during the active phase (Days 3-9) as assessed by the Postoperative Nausea and Vomiting scale

Question	Xanomeline Alone (*n* = 33)		Xanomeline + Trospium (*n* = 35)	
Result	*N*[a]	*n* (%)	*N*[a]	*n* (%)
Have you experienced a feeling of nausea (an unsettled feeling in the stomach and slight urge to vomit) during the past 24 hours?	32		34	
Not at all		24 (75.0)		29 (85.3)
Yes, at least once		8 (25.0)		5 (14.7)
Have you vomited or had dry-retching during the past 24 hours?	32		34	
Not at all		27 (84.4)		33 (97.1)
Yes, at least once		5 (15.6)		1 (2.9)

[a]Analyzed in the evaluable population in subjects with ≥1 result reported during the active phase (Days 3–9)

Table S2 Patient-reported salivation during the active phase (Days 3–9) as assessed by the Unified Parkinson's Disease Rating Scale salivation subscale

During the past 24 hours, please rate your degree of salivation[a]	Xanomeline Alone (*n* = 33)		Xanomeline + Trospium (*n* = 35)	
	N[b]	*n* (%)	*N*[b]	*n* (%)
Day 3	32		34	
Not present		27 (84.4)		31 (91.2)
Present		5 (15.6)		3 (8.8)
Day 4	32		34	
Not present		24 (75.0)		28 (82.4)
Present		8 (25.0)		6 (17.6)
Day 5	32		33	
Not present		26 (81.3)		32 (97.0)
Present		6 (18.8)		1 (3.0)
Day 6	31		33	
Not present		26 (83.9)		29 (87.9)
Present		5 (16.1)		4 (12.1)
Day 7	31		33	
Not present		26 (83.9)		30 (90.9)
Present		5 (16.1)		3 (9.1)
Day 8	31		33	
Not present		27 (87.1)		31 (93.9)
Present		4 (12.9)		2 (6.1)
Day 9	31		31	
Not present		26 (83.9)		30 (96.8)
Present		5 (16.1)		1 (3.2)

[a]Unified Parkinson's Disease Rating Scale salivation subscale assessed using a 5-point scale of 0 (“none”), 1 (“slight but definite excess of saliva in mouth, may have nighttime drooling”), 2 (“moderately excessive saliva, may have minimal drooling”), 3 (“marked excess of saliva with some drooling”), and 4 (“marked drooling, requires constant tissue or handkerchief”). Salivation “not present” was defined as score of 0; salivation “present” was defined as scores of 1, 2, 3, or 4

[b]Analyzed in the evaluable population in subjects with a reported result at the specified visit

Table S3 Patient-reported sweating during the active phase (Days 3–9) as assessed by the Hyperhidrosis Disease Severity Scale

During the past 24 hours, how would you rate the severity of your sweating?[a]	Xanomeline Alone (*n* = 33)		Xanomeline + Trospium (*n* = 35)	
	N[b]	*n* (%)	*N*[b]	*n* (%)
Day 3	32		34	
Not present		27 (84.4)		33 (97.1)
Present		5 (15.6)		1 (2.9)
Day 4	32		34	
Not present		24 (75.0)		32 (94.1)
Present		8 (25.0)		2 (5.9)
Day 5	32		33	
Not present		24 (75.0)		33 (100.0)
Present		8 (25.0)		0
Day 6	31		33	
Not present		21 (67.7)		31 (93.9)
Present		10 (32.3)		2 (6.1)
Day 7	31		33	
Not present		24 (77.4)		32 (97.0)
Present		7 (22.6)		1 (3.0)
Day 8	31		33	
Not present		26 (83.9)		32 (97.0)
Present		5 (16.1)		1 (3.0)
Day 9	31		31	
Not present		25 (80.6)		30 (96.8)
Present		6 (19.4)		1 (3.2)

[a]Hyperhidrosis Disease Severity Scale assessed using a 4-point scale of 1 (“my sweating is never noticeable and never interferes with my daily activities”), 2 (“my sweating is tolerable but sometimes interferes with my daily activities”), 3 (“my sweating is barely tolerable and frequently interferes with my daily activities”), and 4 (“my sweating is intolerable and always interferes with my daily activities”). Sweating “not present” was defined as score of 1; sweating “present” was defined as scores of 2, 3, or 4

[b]Analyzed in the evaluable population in subjects with a reported result at the specified visit

Table S4 Bristol Stool Form Scale

Please rate your stool over the past 24 hours according to the "type"[a]	Xanomeline Alone (n = 33)		Xanomeline + Trospium (n = 35)	
	N[b]	*n* (%)	*N*[b]	*n* (%)
Day 1	24		23	
Type 1		1 (4.2)		0
Type 2		5 (20.8)		4 (17.4)
Type 3		7 (29.2)		10 (43.5)
Type 4		7 (29.2)		8 (34.8)
Type 5		3 (12.5)		0
Type 6		1 (4.2)		1 (4.3)
Type 7		0		0
Day 2	26		28	
Type 1		2 (7.7)		1 (3.6)
Type 2		3 (11.5)		4 (14.3)
Type 3		8 (30.8)		13 (46.4)
Type 4		9 (34.6)		7 (25.0)
Type 5		1 (3.8)		2 (7.1)
Type 6		3 (11.5)		0
Type 7		0		1 (3.6)
Day 3	29		26	
Type 1		0		2 (7.7)
Type 2		6 (20.7)		0
Type 3		11 (37.9)		14 (53.8)
Type 4		5 (17.2)		7 (26.9)
Type 5		4 (13.8)		2 (7.7)
Type 6		1 (3.4)		0
Type 7		2 (6.9)		1 (3.8)
Day 4	20		19	
Type 1		1 (5.0)		3 (15.8)
Type 2		4 (20.0)		1 (5.3)
Type 3		5 (25.0)		8 (42.1)
Type 4		5 (25.0)		5 (26.3)
Type 5		4 (20.0)		1 (5.3)

Type 6		1 (5.0)		0
Type 7		0		1 (5.3)
Day 5	26		21	
Type 1		2 (7.7)		2 (9.5)
Type 2		3 (11.5)		1 (4.8)
Type 3		7 (26.9)		6 (28.6)
Type 4		8 (30.8)		7 (33.3)
Type 5		5 (19.2)		4 (19.0)
Type 6		1 (3.8)		1 (4.8)
Type 7		0		0
Day 6	29		27	
Type 1		0		2 (7.4)
Type 2		4 (13.8)		4 (14.8)
Type 3		9 (31.0)		10 (37.0)
Type 4		10 (34.5)		7 (25.9)
Type 5		5 (17.2)		2 (7.4)
Type 6		0		1 (3.7)
Type 7		1 (3.4)		1 (3.7)
Day 7	28		31	
Type 1		2 (7.1)		2 (6.5)
Type 2		5 (17.9)		4 (12.9)
Type 3		8 (28.6)		10 (32.3)
Type 4		7 (25.0)		10 (32.3)
Type 5		5 (17.9)		3 (9.7)
Type 6		1 (3.6)		1 (3.2)
Type 7		0		1 (3.2)
Day 8	28		30	
Type 1		3 (10.7)		1 (3.3)
Type 2		6 (21.4)		7 (23.3)
Type 3		8 (28.6)		7 (23.3)
Type 4		9 (32.1)		8 (26.7)
Type 5		2 (7.1)		5 (16.7)
Type 6		0		0

Type 7		0		2 (6.7)
Day 9	27		27	
Type 1		4 (14.8)		2 (7.4)
Type 2		6 (22.2)		3 (11.1)
Type 3		4 (14.8)		8 (29.6)
Type 4		7 (25.9)		9 (33.3)
Type 5		5 (18.5)		3 (11.1)
Type 6		0		1 (3.7)
Type 7		1 (3.7)		1 (3.7)
Day 10/early termination	22		27	
Type 1		2 (9.1)		1 (3.7)
Type 2		6 (27.3)		4 (14.8)
Type 3		4 (18.2)		9 (33.3)
Type 4		8 (36.4)		7 (25.9)
Type 5		1 (4.5)		4 (14.8)
Type 6		0		1 (3.7)
Type 7		1 (4.5)		1 (3.7)

[a]Bristol Stool Form Scale assessed using a 7-point scale of Types 1 (“separate hard lumps, like nuts”), 2 (“sausage-shaped but lumpy”), 3 (“like a sausage or snake but with cracks on its surface”), and 4 (“like a sausage or snake, smooth and soft”), 5 (“soft blobs with clear-cut edges”), 6 (“fluffy pieces with ragged edges, a mushy stool”), and 7 (“watery, no solid pieces”). Types 1–2 are typically classified as “constipation,” types 3–5 as “normal,” and types 6–7 as “diarrhea” (Longstreth et al. 2006)

[b]Analyzed in the evaluable population in individuals with a reported result at the specified visit

REFERENCES

Longstreth GF, Thompson WG, Chey WD, Houghton LA, Mearin F, Spiller RC (2006) Functional bowel disorders. Gastroenterology 130:1480-1491 10.1053/j.gastro.2005.11.061
